# Choroid plexus volume in pathology-confirmed Alzheimer's disease

**DOI:** 10.1016/j.tjpad.2026.100648

**Published:** 2026-08-11

**Authors:** Francis Fernandes, Avyarthana Dey, Andy Ma, Nathan W. Churchill, Corinne E. Fischer, Simon J. Graham, David G. Munoz, Tom A. Schweizer

**Affiliations:** a Keenan Research Centre for Biomedical Science, Li Ka Shing Knowledge Institute - St. Michael’s Hospital, Toronto, Ontario, Canada; b Institute of Medical Sciences, Temerty Faculty of Medicine - University of Toronto, Toronto, Ontario, Canada; c Department of Physics - Toronto Metropolitan University, Toronto, Ontario, Canada; d Physical Sciences Platform - Sunnybrook Research Institute, Toronto, Ontario, Canada; e Department of Medical Biophysics, Temerty Faculty of Medicine – University of Toronto, Toronto, Ontario, Canada; f Department of Laboratory Medicine & Pathobiology, Temerty Faculty of Medicine - University of Toronto, Toronto, Ontario, Canada; g Division of Pathology - St. Michael’s Hospital, Toronto, Ontario, Canada; h Division of Neurosurgery, Temerty Faculty of Medicine - University of Toronto, Toronto, Ontario, Canada

**Keywords:** Alzheimer's disease, Choroid plexus volume, Cognitive impairment, Post-mortem Alzheimer's pathology, Magnetic Resonance Imaging

## Abstract

**Introduction:**

The choroid plexus (CP) increases in volume across the Alzheimer’s disease (AD) continuum, suggesting its potential as a clearance-related biomarker. However, few studies have examined *ante-mortem* CP volume in relation to *post-mortem* AD pathology, the gold standard for diagnosis.

**Methods:**

Participants who had structural magnetic resonance imaging and post-mortem pathology, with an interval of ≤ 5 years between imaging and death, were examined. Normalized CP volume (NCPV) was semi-automatically segmented from the lateral ventricles and analyzed using Bayesian linear regression to estimate associations with cognitive impairment (CI), AD pathology, and relevant clinical/demographic data.

**Results:**

Intermediate and high levels of AD pathology and CI were associated with larger NCPV, whereas female sex was associated with lower NCPV. Subgroup analyses showed larger NCPV in individuals with greater CI despite comparable levels of AD pathology.

**Discussion:**

These findings link CP enlargement to neuropathologically confirmed AD burden and CI, supporting further investigation of CP structure and function in AD.

## 1 Introduction

Alzheimer's disease (AD) is a progressive neurodegenerative disorder and the leading cause of dementia worldwide. The onset and progression of AD is pathologically characterized by the accumulation of amyloid-β (Aβ) plaques and neurofibrillary tangles [1]. Increasing evidence suggests that impaired clearance of these proteins, rather than their overproduction alone, plays a critical role in disease onset and progression [2]. Multiple clearance pathways have been implicated in AD, including transport across the blood–brain and blood–cerebrospinal fluid (CSF) barriers, intramural periarterial drainage, and the glymphatic system. Among these, CSF dynamics are thought to play an integral role, with disruptions in CSF production and/or flow contributing to the accumulation of AD pathology [3,4].

The choroid plexus (CP) is a highly vascularized epithelial structure located within the brain ventricles, which serves as the primary source of CSF production [[5], [6], [7], [8]]. Beyond CSF secretion, the CP is essential for formation of the blood-CSF barrier [9], immune surveillance [8], and active clearance of neurotoxic compounds from the CSF [10]. In AD, both the structure and function of the CP appear to be compromised, leading to alterations in secretory, barrier, and transport functions [6]. Such dysfunction has been linked to reduced Aβ clearance and impaired CSF turnover, processes that may contribute to downstream pathological accumulation. Preclinical studies have demonstrated a key role for the CP in Aβ removal and have further shown that this function is disrupted in AD models [11,12]. Consistent with these findings, *post-mortem* studies in individuals with AD have reported pronounced morphological alterations of the CP, including structural remodelling and accumulation of aberrant metabolites [6,13,14].

Neuroimaging studies have further examined CP volume across the cognitive continuum. Collectively, these studies show increased CP volume in individuals with dementia compared with cognitively normal controls [[15], [16], [17], [18], [19]]. Similarly, the onset and progression of CI in the AD spectrum has also been linked to increased CP volumes [6,15,16,18]. Elevated CP volume has also been linked to other established neuroimaging markers of AD pathology, including amyloid & tau positivity on positron emission tomography, hippocampal atrophy, and ventricular enlargement [[19], [20], [21]].

Despite these convergent lines of research, few studies have directly examined the relationship between *in vivo* CP volume and neuropathologically confirmed AD, the gold standard for diagnosis. The present study examined whether CP volume, measured during life, is associated with (1) the severity of *post-mortem* AD pathology and (2) the degree of CI. It was hypothesized that greater AD pathology burden and more severe CI are each associated with increased CP volume. To further clarify the relationship between CP volume and cognition, CP volume was also examined across individuals with comparable levels of AD pathology but differing levels of CI. It was hypothesized that, even within similar pathological strata, higher CP volume is associated with worse CI.

## 2 Methods

### 2.1 Source population

A retrospective analysis was conducted on patient data collected from the National Alzheimer's Coordinating Center (NACC), which included both clinical assessments and MRI data. Briefly, the NACC consists of multiple institute-funded AD research centers across the United States responsible for recruiting and collecting data on participants across the aging and AD spectrum. These participants make up the NACC Uniform Data Set (UDS), which contains data across multiple patient domains including standardized clinical, demographic and cognitive data [22,23]. A subset of these participants also underwent structural magnetic resonance imaging (MRI), as described below (see MRI Acquisition & Processing).

Neuropathological data were obtained from the NACC Neuropathology Data Set (NDS), which includes standardized *post-mortem* assessments of multiple neuropathological features, including Alzheimer’s disease-related neuropathological change (ADNC) [24]. Data from the UDS and NDS were merged and assessed for eligibility. The UDS, NDS and associated neuroimaging data is obtainable from the NACC via request.

Informed consent was obtained from participants and their informants at the contributing Alzheimer’s Disease Research Centers. All study protocols were approved by the University of Washington Institutional Review Board. Detailed descriptions of the data collection procedures, instruments, and variable definitions for the UDS and NDS have been published previously [25].

## 3 Inclusion & exclusion criteria

UDS and NDS data available as of September 2024 were examined for eligibility. Inclusion criteria were: (1) availability of neuropathological data, including classification of ADNC; (2) availability of a 3 Tesla T1-weighted MRI; (3) an interval of ≤ 5 years between T1-weighted MRI acquisition and death/*post-mortem* examination; (4) age > 60 years at the time of MRI; and (5) availability of clinician-rated cognitive status at the study visit corresponding to the MRI.

To restrict analyses to late-onset AD, participants with primary neuropathological diagnoses other than AD were excluded. These included frontotemporal lobar degeneration and related subtypes (*e.g.,* Pick’s disease, corticobasal degeneration, progressive supranuclear palsy, *etc.*), multiple system atrophy, prion disease, trinucleotide repeat disorders, neoplasms, primary white matter disease, and infectious processes (abscess, encephalitis, *etc.*). Definitions, identification criteria, and staging procedures for these pathologies are described in the NACC Neuropathology Data Set [24].

Given that AD pathology frequently co-occurs with other neurodegenerative and aging-related pathologies [26], participants with ADNC were retained in the sample if comorbid cerebrovascular pathology, Lewy body pathology, age-related tau astrogliopathy (ARTAG), and/or TAR DNA-binding protein 43 (TDP-43) pathology were present. Participants meeting these criteria were subsequently assessed for MRI quality.

## 4 Pathological assessments

Neuropathological reporting of AD (obtained from the NDS) was reported using semi-quantitative ABC criteria consisting of Thal staging (criteria A) [30], Braak staging (criteria B) [27] and the Consortium to Establish a Registry for Alzheimer’s Disease (CERAD) (criterion C) [28].

Together, these criteria were combined to classify ADNC burden as “Not ADNC”, “Low”, “Intermediate” and “High”. Definitions for each pathological stage are described elsewhere [29].

For analyses in this study, the “Not” and “Low” ADNC categories were combined and referred to as “Slight ADNC.” This grouping was implemented to account for the relatively small number of participants with low pathological burden compared with the Intermediate and High ADNC groups.

## 5 Clinical assessment

Cognitive status was assessed based on clinician-determined criteria described in the UDS [25]. In brief, normal cognition was defined as follows: global Clinical Dementia Rating scale = 0 [30]; neuropsychological testing within normal ranges and normal behaviour. Mild CI (MCI) was defined as: clinician-observed impairment in one or more cognitive domains (*e.g.,* memory, language, executive function, and visuospatial skills); concern (by clinician or co-participant) of a change in cognition from previous levels; and currently preserved independence in functional abilities. Dementia was defined as: presence of cognitive and/or behavioural symptoms, decline in previous levels of functioning; interference with the ability to function at usual activities and/or work; CI detected/diagnosed through previous clinical history and not explained by delirium or another major psychiatric disorder. Across the sample, available instances of the completed Mini-Mental Status Examination (MMSE) [31] were also examined for exploratory sub-analysis.

Considering the vascular involvement of the CP [19,32,33], cardiovascular risk factors were also assessed. Body mass index (BMI), presence of hypertension and presence of hypercholesterolemia were extracted from the UDS. Hypertension and hypercholesterolemia were reported categorically as either recent/active, remote/inactive or absent. Additionally, impaired sleep, given its reported association with relevant clearance mechanisms in AD [34], [35] and CP-related dysfunction [[36], [37], [38]], was also examined. Sleep disturbance was operationalized as present or absent based on the Neuropsychiatric Inventory Questionnaire (NPI-Q), an informant-based instrument assessing the presence and severity of neuropsychiatric symptoms [39]. All clinical and demographic variables were obtained from the participant visit corresponding to MRI acquisition and therefore were collected within ≤ 5 years of death and *post-mortem* neuropathological assessment.

## 6 MRI acquisition & processing

T1-weighted MRI scans were acquired using multiple imaging protocols and MRI systems across participating AD research centers. All images included in the present study underwent independent manual quality assessment by two raters to evaluate image suitability for analysis, including the presence of motion artifacts, focal brain lesions or injuries, and anatomical abnormalities that could compromise image processing (per criteria provided by Backhausen and colleagues [40]). Image inspection was performed using an MRI viewer (Insight Segmentation and Registration Toolkit, ITK-SNAP) [41]. See APPENDIX 1 for further information about visual inspection. Structural images that passed quality control were processed using FreeSurfer (version 6.0). The standard *recon-all* pipeline was applied to resample images to 1 mm isotropic voxel resolution and to perform automated atlas-based cortical and subcortical parcellation using the Desikan–Killiany atlas [42].

### 6.1 CP segmentation

**Fig. 1:**
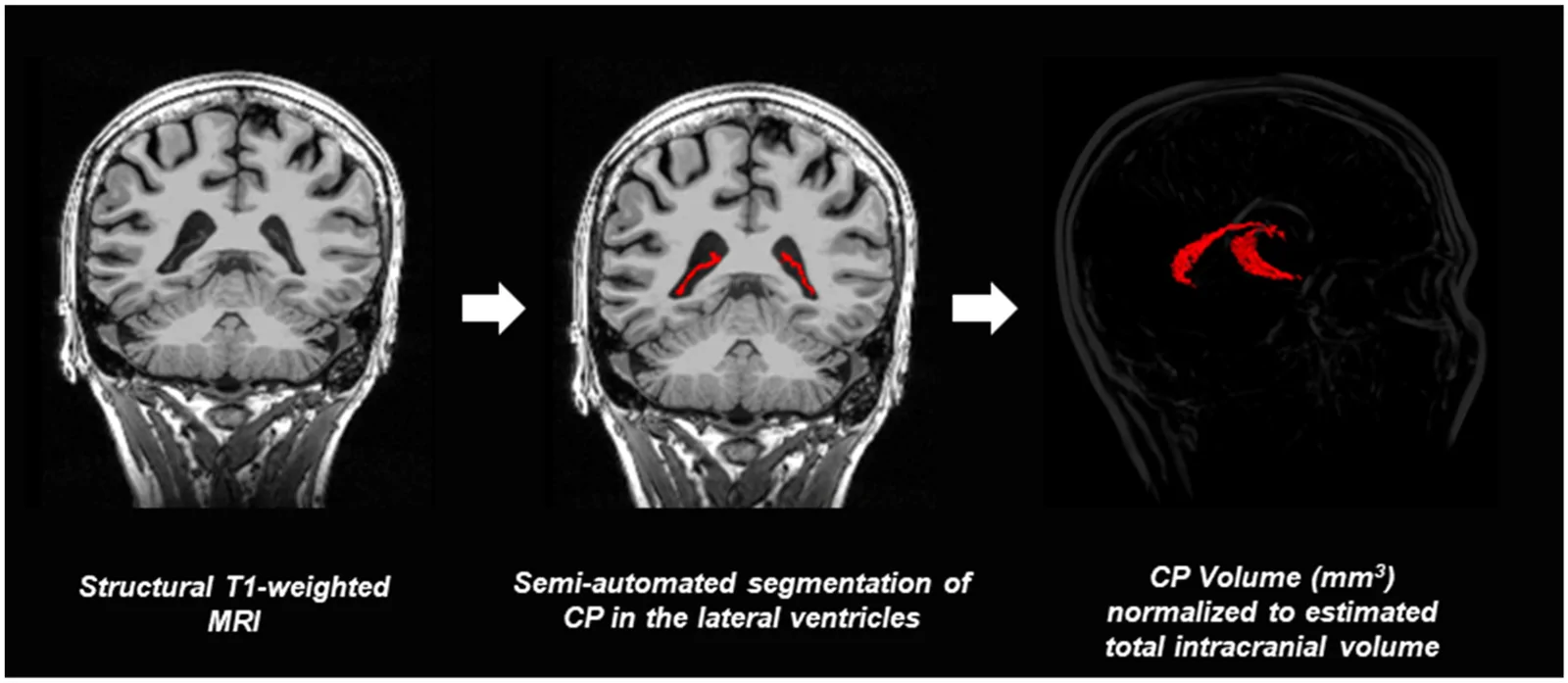
Semi-Automated Segmentation of the Choroid Plexus from T1-weighted images. A Gaussian Mixture Model [20] was used to automatically parcellate the CP within the lateral ventricles (using FreeSurfer-derived masks). The resulting segmentation was then manually corrected using ITK-SNAP [41] and normalized to the FreeSurfer-estimated total intracranial volume. Magnetic Resonance Imaging (MRI), Choroid Plexus (CP), Insight Segmentation and Registration Toolkit (ITK).

Estimated total intracranial volume (eTIV) and the CP volume from the lateral ventricles were obtained using the FreeSurfer recon-all pipeline. Although FreeSurfer provides automated CP segmentation, this approach has been shown to be inaccurate in some cases [20]. To improve segmentation accuracy, a lightweight Gaussian Mixture Model (GMM)–based algorithm was applied to the FreeSurfer-derived CP segmentation, an approach that has demonstrated improved performance relative to FreeSurfer alone [20] .All resulting CP segmentations were subsequently manually reviewed and corrected by a single rater, blinded to the clinical/pathological characteristics of each participant, to address cases of under- or over-segmentation using the Insight Segmentation and Registration Toolkit (ITK-SNAP) [41] (Fig. 1). Manual corrections involved the systematic inspection of voxels adjacent to the lateral ventricles that may have been incorrectly labeled as CP. This included neighboring white and gray matter structures such as the body and splenium of the corpus callosum, fornix, hippocampus, thalamus, vertical portion of the caudate nucleus, and surrounding cerebral white matter (see APPENDIX 1 for more information). Final CP volumes were calculated from the corrected segmentations and divided by eTIV to provide a normalized CP volume (NCPV) which accounts for interindividual differences in head size.

### 6.2 Statistical analysis

To examine the potential effects of cognition and AD path burden on NCPV, a Bayesian linear regression model was employed. Compared with more commonly used frequentist methods, the Bayesian framework allows for intuitive interpretation of results by reporting the probability of an effect for the proposed model, given the observed data. This framework integrates relevant priors and model likelihoods within a single flexible estimation process, without the strict assumptions (*e.g.*, sample size, normality, homoscedasticity) that are often required of frequentist linear regression models. The use of weakly informative priors also helps to constrain effect sizes to plausible ranges and to stabilize estimates in smaller subgroups. Together, these features make the Bayesian framework well suited for examining subtle relationships among NCPV, AD pathology, and cognition.

Models were fit using Hamiltonian Monte Carlo sampling implemented in the *brms* package in R (version 4.3.1), with four chains of 4000 iterations each (including warm-up). Posterior convergence was assessed using standard diagnostic checks and visual inspection of sampling traces. Posterior draws were processed and visualized using *tidybayes* and *ggplot2*. Posterior distributions were summarized using posterior medians; 90% highest density intervals (HDIs); and the probability of direction (PD), defined as the posterior probability that an effect was greater (or less) than zero. Group-level marginal means and pairwise contrasts for categorical predictors were computed using *emmeans* extended to the Bayesian framework. Based on research aims and hypotheses, Bayesian linear regression models were specified with normally distributed likelihoods and weakly informative priors. Two models were implemented.

The first model evaluated the effects of ADNC, cognitive status, presence or absence of CVD risk factors, sleep disturbances, age and sex on NCPV:

(1) $$\text{Normalized}\;\text{CP}\;\text{Volum}e_{i}=\beta_{1}\left(\text{AD}\;\text{Pathology}\;\text{Burde}n_{i}\right)+\beta_{2}\left(\text{Cognitive}\;\text{Statu}s_{i}\right)+\beta_{3}\left(\text{Ag}e_{i}\right)+\beta_{4}\left(\text{Se}x_{i}\right)+\beta_{5}\left(\text{CVD}\;\text{Risk}\;\text{Factor}s_{i}\right)+\beta_{6}\left(\text{Sleep}\;\text{Disturbance}s_{i}\right),$$

where *i* indexes the participants and coefficients β_1_- β_6_ represent the regression coefficients for each predictor in the model. Similarly, the second model was designed to assess the correlates of cognitive test performance (via the MMSE scores) in AD among participants with comparable levels of AD pathology:

(2) $$\text{Normalized}\;\text{CP}\;\text{Volum}e_{i}=\beta_{1}\left(\text{MMS}E_{i}\right)+\beta_{2}\left(\text{Ag}e_{i}\right)+\beta_{3}\left(\text{Se}x_{i}\right)+\beta_{4}\left(\text{CVD}\;\text{Risk}\;\text{Factor}s_{i}\right)+\beta_{5}\left(\text{Sleep}\;\text{Disturbance}s_{i}\right).$$

Here, age, sex, the presence or absence of CVD risk factors and the presence or absence of sleep disturbances were modelled alongside MMSE scores to gauge the relationship to CI within MCI and dementia cohorts. Participants for this analysis were restricted to having high ADNC burden, controlling for additional variability in AD pathology while while retaining sufficient observations for exploratory subgroup analyses..

NCPV and non-categorical predictors were standardized (via Z-scoring - subtracting the sample mean and dividing by the sample standard deviation) before model fitting to place parameters on a common scale. Based on the distribution of these standardized values, weakly informative Normal priors were placed on regression coefficients (β∼N(0,2.5)). Default *brms* Student-t priors were used for the intercept (centered on the outcome mean in each analysis subset) and a half-Student-t prior for the residual standard deviation. For this study, the threshold for a credible effect was set at PD ≥ 85%. To ensure adequate sample representation, group comparisons were restricted to cognitive-pathological categories with ≥ 5 participants (Table 1). Posterior medians, HDIs and corresponding PD values for all variables are provided in the Supplementary Materials.

**Table 1:** Clinical, Pathological & Imaging Characteristics across the Sample.

**Clinical & Imaging Characteristics**	**AD Pathological Burden**		
	**Slight (n = 18)**	**Intermediate (n = 23)**	**High (n = 70)**
Age at Death (mean ± SD)	78.6 ± 9	83.7 ± 5.7	77.2 ± 8.2
Females (n, % in pathology group)	4 (22%)	9 (39%)	26 (37%)
Active Hypertension (n, % in pathology group)	7 (39%)	9 (39%)	28 (40%)
Active Hypercholesterolemia (n, % in pathology group)	6 (33%)	13 (57%)	27 (39%)
Body Mass Index (mean ± SD)	28.6 ± 4.4	25.6 ± 3.8	26.1 ± 4.4
Presence of Sleep Disturbance (n, % in pathology group)	5 (28%)	5 (22%)	19 (27%)
**Clinician-determined Cognitive Status**			
Cognitively Intact (n, % in pathology group)	12 (67%)	6 (26%)	5 (7%)
Mild Cognitive Impairment (n, % in pathology group)	2 (11%)	11 (48%)	17 (24%)
Dementia (n, % in pathology group)	4 (22%)	6 (26%)	48 (69%)
**Imaging Findings Z Score**			
Normalized Choroid Plexus Volume (mean ± SD)	−0.58 ± 1.00	0.1 ± 1.07	0.11 ± 0.96

Values are expressed as mean ± standard deviation or count (percentage) within AD pathological burden group (slight, intermediate, high).

## 7 Results

**Fig. 2:**
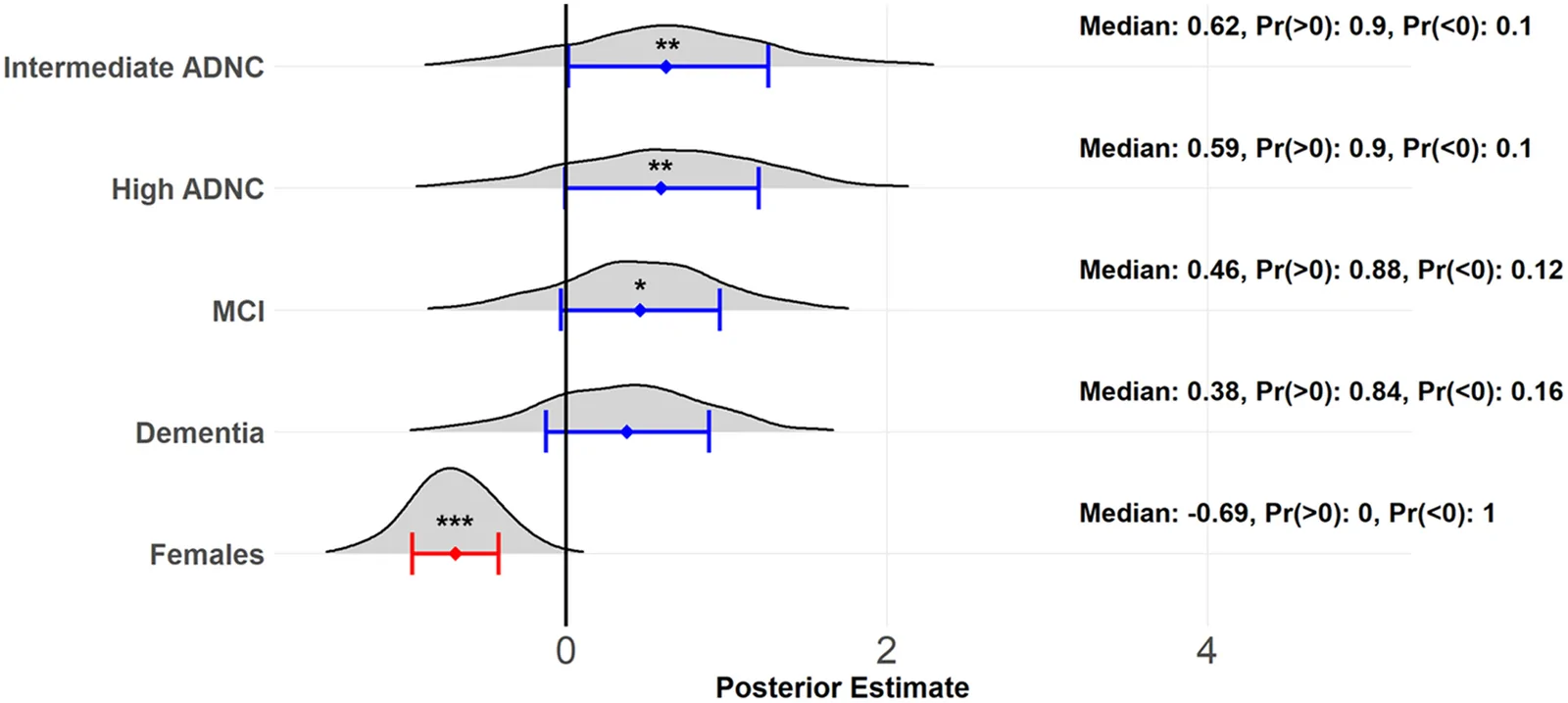
Bayesian Modelling of the Sex, Pathology & Cognition on Choroid Plexus Volume. Credible effects (PD ≥ 85%) of AD pathology (intermediate & high ADNC), MCI and female sex on NCPV were observed. Effect sizes are reported as median values and the probability of direction is reported as PD. * for PD of 85%−89%, ** for PD of 90%−94%, *** for PD >95%. Alzheimer’s Disease Neuropathological Change (ADNC), Alzheimer’s Disease (AD), Probability of Direction (PD), Normalized Choroid Plexus Volume (NCPV), Mild Cognitive Impairment (MCI).

The final sample consisted of 111 participants, for whom NCPV values were evaluated in relation to cognition, AD-related pathology and other demographic and clinical variables (Table 1). Participants were stratified into three AD pathological burden groups: slight (n = 18), intermediate (n = 23), and high (n = 70). Age at death did not differ markedly between groups, with mean values ranging from 77 to 84 years. Female representation was lower in the slight pathology group (22%) than in intermediate (39%) and high (37%) pathology groups. The prevalence of CI (*i.e.,* the presence of MCI or dementia) increased with higher AD pathology burden, from 33% in slight ADNC to 93% in high ADNC. Additional demographic and clinical variables are summarized in Table 1. Results for the most salient findings of CP-related effects are summarized in Fig. 2, with additional distributions, effect sizes and comparisons listed in the Supplementary Materials.

### 7.1 Main effects of AD pathology and cognitive impairment on CP volume

**Fig. 3:**
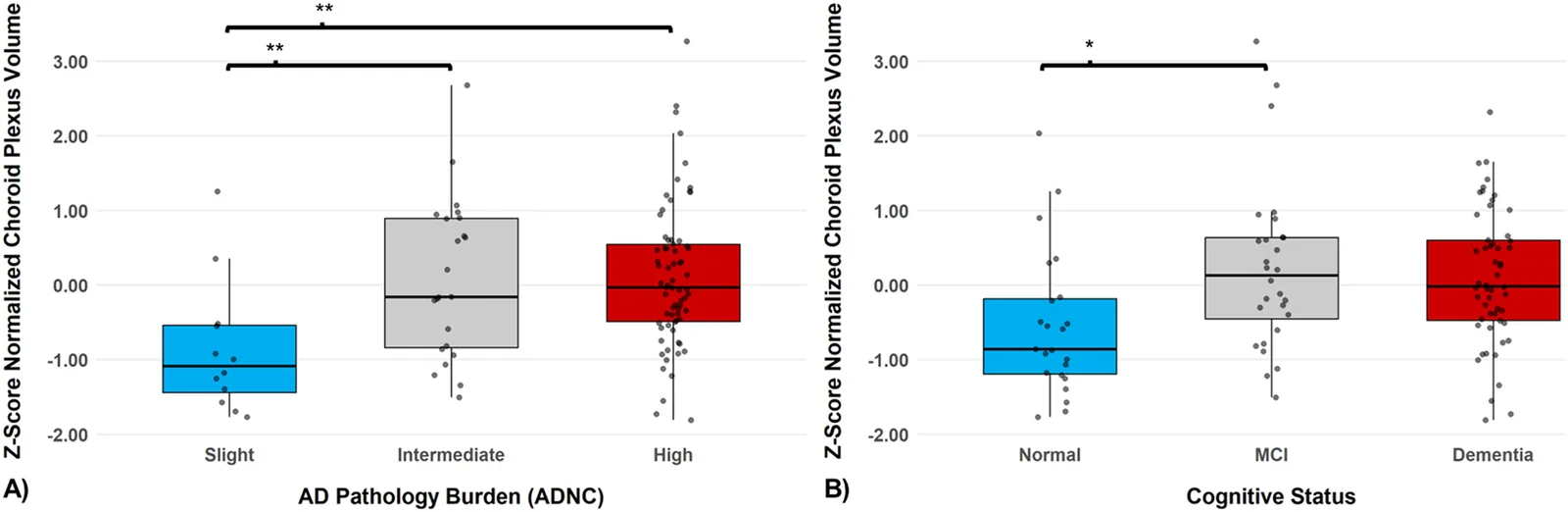
Group-level Comparisons of Normalized Choroid Plexus Volume across Pathology & Cognition. A) Group-level comparisons across increasing AD pathological burden, categorized by ADNC status. B) Group-level comparisons across increasing CI, categorized via clinician-determined cognitive status. * for PD of 85%−89%, ** for PD of 90%−94%, *** for PD >95%. Alzheimer’s Disease Neuropathological Change (ADNC), Alzheimer’s Disease (AD), Cognitive Impairment (CI), Mild Cognitive Impairment (MCI).

The NCPV was found to be increased in individuals with intermediate ADNC (median = 0.63, 90% HDI [0.009] – [1.26], PD = 90%) and high ADNC (median = 0.59, 90% HDI [−0.008] – [1.20], PD = 90%) compared to those with slight ADNC (Fig. 2). Pairwise comparisons showed no credible differences between high and intermediate ADNC (median = 0.04, 90% HDI [−0.30] – [0.38], PD = 56%) (Fig. 3A).

Additionally, individuals with MCI showed higher NCPV (median = 0.461, 90% HDI [−0.03] – [0.96], PD = 88%) when compared to cognitively intact individuals (Fig. 2). Participants with dementia showed increased NCPV compared to cognitively intact individuals though the probability of direction did not meet credibility thresholds (median = 0.38, 90% HDI [−0.13] – [0.89], PD = 84%) Pairwise comparisons between MCI and dementia showed no credible differences in NCPV (median = 0.08, 90% HDI [−0.24] – [0.41], PD = 63%) (Fig. 3B). Biological sex exhibited the largest median effect on NCPV where females had lower NCPVs compared to males (median = −0.69, 90% HDI [−0.962] – [−0.42], PD > 99%). Age, CVD factors and sleep did not show any credible effects on NCPV across the overall sample (see Supplementary Materials for effect sizes and probability estimates; S2-S4). To further interrogate observations, sensitivity analyses were also performed (Supplementary Materials; S7-S12 & S15-S22, S24-S27). This included: exploring alternate model priors; comparing results to more simplified regression models; and modelling the effects of additional variables on CP volumes such as time interval between MRI and death, individual effects of co-pathologies, severity of co-pathologies (*i.e.,* CAA) and lateral ventricular volume as a covariate.

Summarizing the results of these sensitivity analyses, the observed effects for intermediate ADNC, high ADNC, females and MCI were mostly retained with minor variations in effect sizes and PD ≥ 85% across sensitivity analyses involving altered priors and the addition of the interval between MRI and death as a covariate. Similar findings were observed when individually assessing the presence of CAA, TDP-43, ARTAG and Lewy bodies (See S15, S18-S20 in Supplementary Materials).

### 7.2 Exploring CP volume with varying cognitive impairment in high AD pathology

A subset of participants (n = 10 with MCI, n = 23 with dementia) with available MMSE scores and high ADNC were further examined in terms of associations between NCPV and CI in a cohort with comparable burden of AD pathology. Among participants with MCI and high ADNC (Table 2), no credible associations were observed between NCPV and MMSE scores (median = −0.03, 90% HDI [−0.24] – [0.11], PD = 64%).

**Table 2:** Modelling the Association between NCPV and Varying CI within a Sub-group of MCI with High AD Pathology.

**NCPV & Effect of Varying CI in Participants with MCI with High AD Pathology (n = 10)**							
**Parameter**	**Age at Death**	**MMSE Score**	**Females**	**Body Mass Index**	**Active Hypertension**	**Active Hypercholesterol-emia**	**Active Sleep Disturbances**
**Descriptive Statistics**	79.1 yrs ± 7.3	27 ± 2.1	3 (30%)	27.4 ± 3.3	5 (50%)	4 (40%)	1 (10%)
**Effect Size (PD)**	−8.5 × 10–3 (56%)	-0.03 (64%)	**−3.76 (97%)**	0.2 (76%)	**−2.2 (96%)**	**−0.55 (90%)**	**1.**27 **(87%)**

Descriptive statistics are expressed as mean ± standard deviations for continuous variables and counts (percentages) for categorical. Effect sizes are reported as posterior medians, with corresponding probabilities reported as PD. Bolded effect size values indicate credible findings (PD ≥ 85%).

Normalized Choroid Plexus Volume (NCPV), Cognitive Impairment (CI), Mini-Mental State Examination (MMSE), Mild Cognitive Impairment (MCI), Alzheimer's Disease (AD), Probability of Direction (PD).

Like the overall sample, biological females exhibited lower NCPV compared to biological males (median = −3.76, 90% HDI [−4.82] – [−1.49], PD = 97%). Participants with recent/active hypertension and hypercholesterolemia also exhibited lower NCPV compared to those with remote/inactive hypertension (median = −2.23, 90% HDI [−3.71] – [−0.94], PD = 96%) and remote/inactive hypercholesterolemia (median = −0.55, 90% HDI [−1.36] – [−0.005], PD = 90%), respectively. The presence of sleep disturbances was also associated with higher NCPV (median = 1.27, 90% HDI [−0.27] – [2.09], PD = 87%). Although these associations showed high probabilities of effect, they should be interpreted cautiously due to the limited sample size.

Twenty-three participants with dementia, high ADNC and reported MMSE scores were similarly modeled (Table 3). In this group, MMSE scores were negatively associated with NCPV such that lower MMSE scores, indicating greater CI, were associated with higher NCPV (median = −0.11, 90% HDI [−0.22] – [0.00], PD = 90%). Older age was also associated with a modest increase in NCPV (median = 0.07, 90% HDI [0.01] – [0.12], PD = 96%). Participants with active hypercholesterolemia demonstrated credible associations with lower NCPV (median = −0.71, 90% HDI [−1.36] – [0.02], PD = 91%). Lastly, sleep disturbances were credibly associated with increased NCPV (median = 0.71, 90% HDI [−0.05] – [1.50], PD = 88%). Although female sex was negatively associated with NCPV (median = −0.46, 90% HDI [−1.33, 0.45], PD = 75%), consistent with the overall results, the effect did not meet the established credibility threshold and was likely attributable to the small sample size.

**Table 3:** Modelling the Association between NCPV and Varying CI within a Sub-group of Dementia with High AD Pathology.

**NCPV & Effect of Varying CI in Participants with Dementia with High AD Pathology (n = 23)**							
**Parameter**	**Age at Death**	**MMSE Score**	**Females**	**Body Mass Index**	**Recent/Active Hypertension**	**Recent/Active Hypercholesterol emia**	**Recent Sleep Disturbances**
**Descriptive Statistics**	74 yrs ± 8.1	21 ± 3.6	4 (17%)	27 ± 4.7	11 (48%)	9 (39%)	6 (26%)
**Effect Size (PD)**	**6.7×10–2** **(96%)**	**−0.11 (90%)**	−0.46 (75%)	−0.02 (59%)	−0.18 (63%)	**−0.71 (91%)**	**0.71 (88%)**

Descriptive statistics are expressed as mean ± standard deviations for continuous variables and counts (percentages) for categorical. Effect sizes are reported as posterior medians, with corresponding probabilities reported as PD.

Normalized Choroid Plexus Volume (NCPV), Cognitive Impairment (CI), Mini-Mental State Examination (MMSE), Mild Cognitive Impairment (MCI), Alzheimer's Disease (AD), Probability of Direction (PD).

## 8 Discussion

The present study explored relationships between CP volume, AD pathology (via ADNC determined *post-mortem*), and CI. Overall, intermediate and high AD pathology were associated with a high probability (PD = 90%) of larger CP volumes. Similarly, participants with MCI also showed a high probability (PD = 88%) of larger CP volumes compared to cognitively intact controls, along with near-credible effects (PD = 84%) of larger CP volumes in the dementia group. Additional exploratory analyses probed the relationships between CP volume and CI among participants with comparable levels of AD pathology. Among participants with dementia and high AD pathology, lower MMSE scores were related to larger CP volumes, indicating that CP enlargement may be more pronounced at more severe levels of CI. Across the overall sample, biological females had smaller CP volumes compared to biological males (PD = 99%), suggesting a sex-based effect despite normalization to eTIV.

With respect to AD pathology, CP volume has been examined in relation to blood biomarkers, CSF measures, and PET. Associations between CP volume and amyloid deposition via PET [15], tau deposition via PET 21, CSF levels of amyloid [43], and plasma biomarker presence of amyloid [44] have been reported. Collectively, these studies support the notion that CP volume is associated with increased AD-related pathology observed *in vivo*; however, these studies largely rely on *ante-mortem* biomarkers of pathology. The present study is the first to explore the relationship between *in vivo* CP volume and *post-mortem* AD pathological burden in the same participants. Both intermediate and high ADNC groups showed a high probability (PD = 90%) of larger CP volume compared to the slight ADNC group, though differences between intermediate and high ADNC were not detected (PD = 56%). A relationship between intermediate ADNC and CP volume suggests that CP volume enlargement may occur early in the development of AD.

In AD and other neurodegenerative diseases, an increase in CP volume is typically interpreted as a marker of CP dysfunction. However, the precise processes underlying the changes in CP volumes remain an active area of investigation. Recent histological analyses of the CP demonstrate aberrant accumulation of inflammatory signals and metabolites among the CP of AD patients compared to older adult controls [6]. The onset and progression of AD have also been observed to cause alterations in CP morphology via epithelial atrophy and thickening of the basement membrane leading to remodelling of the CP tissue [14,45]. The increased aggregation of amyloid and Tau also propagate additional injury-related cascades that further drive the changes in the CP basement layer, causing thickening of the CP basement membrane and irregularities in CP morphology, thereby altering CP function [7]. However, there has yet to be work done to directly relate mechanisms of CP alterations at the cellular level to broader *in vivo* changes in CP volume [7].

In parallel with relationships to AD pathology, several studies report larger CP volumes with greater CI (*i.e.,* MCI and dementia) compared to cognitively normal controls [16,19,46]. Findings from the present study are consistent with these reports. Increased CP volumes were observed in the MCI group and neared credibility thresholds in the dementia group, suggesting a ceiling effect that arises during the onset and progression of CI. The absence of credible differences between the MCI and dementia groups further supports this interpretation.

The accumulation of AD pathology, and the myriad physiological consequences that follow are key factors in the onset and progression of CI [47,48]. However, individuals presenting with CI may harbor varying levels of AD pathology and individuals with similar pathological burden may follow different cognitive trajectories [49,50]. In the context of CP volume, heterogeneity in AD pathology and CI complicates the interpretation of whether CP enlargement primarily reflects pathological burden and/or additional physiological processes that contribute to cognitive decline. To further probe these relationships, additional analyses were conducted in a small subgroup of participants with comparable AD pathological burden but different levels of CI based on their MMSE scores. Among participants with dementia and high AD pathology, poorer cognition (indexed by lower MMSE scores) showed a high probability (PD = 90%) of increased CP volume. However, this relationship was not observed in the MCI group. These findings suggest that CP enlargement may reflect processes contributing to CI beyond AD pathology. However, this interpretation remains strictly speculative given the small subgroup sizes (n = 23 for dementia + high ADNC; n = 10 for MCI + high ADNC) and the limited sensitivity of the MMSE for detecting subtle cognitive deficits in MCI or early dementia [51,52].

Subgroup analyses revealed that sleep and cardiovascular variables demonstrated credible associations with CP volume not observed in the overall study sample. Specifically, the presence of sleep disturbances showed a positive association to CP volume among both the MCI (PD = 87%) and dementia (PD = 88%) sub-groups with high AD pathology. CP volume enlargement in the context of sleep impairments is consistent with emerging research examining the relationships between CP dysfunction, CI, and impaired clearance mechanisms related to sleep disruption [37,38,53]. The present findings, although limited in their generalizability because of the limited sample size, suggest a possible interplay between CP dysfunction, CI, and impaired clearance and supports future work explicitly examining these relationships in larger, more, representative samples.

Recently, CVD risk factors have also been increasingly examined alongside CP volume due to the vascular involvement of the CP [19,32,33]. Current analyses did not observe credible findings between CP volume and BMI, presence of hypertension or the presence of hypercholesterolemia across the overall sample. However, subgroup analyses showed that the presence of hypertension (PD = 96%) and hypercholesterolemia (PD = 90%) did associate with lower CP volumes in the MCI /high ADNC group whereas hypercholesterolemia alone (PD = 91%) was associated with lower CP volumes in the dementia /high ADNC group, calling for further investigation into the potential contributions of CVD risk factors on CP volume across the AD spectrum. While both sleep and CVD showed probable effects towards CP volume, the limited sample size and low prevalence of these variables within the subgroups limit interpretation compared to the findings from the sample at large.

Biological sex showed a highly negative effect on CP volume, despite normalization of CP volume to eTIV. Similar sex-related differences in CP volume have been reported in both cross-sectional and longitudinal studies across the AD continuum [16,54]. In the present study, the sex effect exceeded the median effects for cognitive status and AD pathology, highlighting its importance in evaluating CP physiology in normal aging and AD. However, this finding should be interpreted with caution as the sample was not prospectively matched for either age nor sex. Future studies should consider sex-stratified analyses and alternative normalization strategies beyond eTIV (*e.g.,* lateral ventricular volume, CSF volume, etc.) to assess the robustness of these differences to different study methodologies [55].

A key strength of this study is the direct linkage of *in vivo* CP volume with *post-mortem* pathological burden of AD. Combining *post-mortem* evaluation with *ante-mortem* neuroimaging anchors interpretations in the gold-standard diagnosis of AD [56]. Neuropathological data provide precise information on the severity of AD pathology and co-pathologies that may present with AD-like symptoms but do not meet criteria for AD. Prior studies of CP volume have relied primarily on *ante-mortem* biomarkers, which cannot fully resolve these distinctions and may introduce bias. The use of semi-automated CP segmentation with manual correction also enhanced the accuracy of CP segmentation compared to fully automated pipelines, which have been shown to mistakenly over/under-segment regions of the CP [20]. Furthermore, the use of a conservative window of ≤ 5 years applied between MRI and death also increases confidence that *post-mortem* pathology reflected the disease state at the time of image acquisition [54].

The results of the present study should be interpreted considering several limitations. First, the current sample was unevenly distributed in terms of pathology burden, with a high prevalence of high ADNC (> 60% of sample; see Supplementary Materials for full break down of presence/severity of co-pathologies). This, combined with the convenience sampling used in the NACC dataset may impact the generalizability of the findings. Second, a representative assessment on the presence *and* severity of relevant co-pathologies (CAA, TDP-43, ARTAG, Lewy bodies, etc.) would provide more realistic observations on the influence of pathologies on CP volume, considering AD pathology rarely occurs in isolation [56] (See Supplementary Materials for sensitivity analyses in the presence of co-pathologies). Nevertheless, the present observations provide additional evidence for relationships between AD pathology and CP volume and support future efforts to further disentangle the effect of various neurodegenerative pathologies on CP volume enlargement.

Small sample sizes in various subgroups also limited analyses of CI, pathology and their influence on CP volume. Still, the Bayesian framework enable analyses of this unique dataset by reporting effect sizes and their associated probabilities for chosen parameters on CP volume, which may have gone undetected using strictly frequentist approaches. The current framework and observations still provide new evidence supporting relationships between CP volume, cognition and pathology, further informing future research in this space.

Lastly, the cross-sectional study design limits inference regarding CP volume changes across stages of increasing pathology. Repeated-measures analyses relating CP volume and CI to *post-mortem* pathology may be possible with future NACC releases. Future work incorporating longitudinal measures of CP volume in cognitively normal older adults and individuals at early stages of CI (*e.g.*, subjective CI) would be valuable for characterizing CP volume changes across normal aging and early stages of pathological aging. Additionally, longitudinal studies examining CP volume alongside *ante-mortem* markers of AD pathology (blood-based and/or CSF biomarkers), biomarkers of immune activation, and other relevant neuroanatomical correlates (*i.e.,* ventricular volume) would be helpful in parsing out whether CP-related changes are more sensitive to AD pathology or other physiological processes (*e.g.,* neuroinflammation) implicated in the presence of AD pathology. Given the CP’s role in CSF production and homeostasis, future work examining CP volume and AD pathology alongside multiple measures of CSF dynamics would also provide evidence to interrogate CP volume as a measure of dysfunctional clearance in the aging brain.

## 9 Conclusion

The CP has been observed to increase in volume across the AD continuum. The present findings demonstrate that CP enlargement measured using structural MRI is related to gold-standard AD pathology, clinician-determined cognitive status, and sex. By incorporating *post-mortem* AD burden, these findings contextualize prior observations of CP enlargement and provide additional evidence that changes in CP structure may be tied to underlying pathological accumulation. Future studies integrating longitudinal imaging and more direct measures of CSF dynamics will be critical for elucidating the mechanistic underpinnings of CP volume changes and their potential relevance to brain clearance mechanisms.

## Data statement

Data from the National Alzheimer’s Coordinating Center is publicly available to investigators through a data request process at https://naccdata.org.

## Funding sources

None.

## Disclosures/conflict of interest

The authors declared no potential conflicts of interest with respect to the research, authorship, and/or publication of this article.

## Consent statement

Research using the NACC data was approved by the University of Washington Institutional Review Board. All contributing AD research centers obtained informed consent from their participants and received institutional review board approval before submitting data to NACC. Research was conducted in accordance with the Declaration of Helsinki.

## Declaration of generative artificial intelligence use

N/A

## CRediT authorship contribution statement

**Francis Fernandes:** Writing – review & editing, Writing – original draft, Visualization, Methodology, Investigation, Formal analysis, Conceptualization. **Avyarthana Dey:** Writing – review & editing. **Andy Ma:** Software, Methodology. **Nathan W. Churchill:** Writing – review & editing, Methodology. **Corinne E. Fischer:** Writing – review & editing, Conceptualization. **Simon J. Graham:** Writing – review & editing, Methodology. **David G. Munoz:** Writing – review & editing, Conceptualization. **Tom A. Schweizer:** Writing – review & editing, Supervision, Funding acquisition, Conceptualization.

## Declaration of competing interest

The authors declare that they have no known competing financial interests or personal relationships that could have appeared to influence the work reported in this paper.

## Appendix Supplementary materials

Image, application 1

Image, application 2
