# Supplementary material for: Choroid plexus volume in pathology-confirmed Alzheimer's disease
Source: J Prev Alzheimers Dis. 2026 Aug 11;13(9):100648. doi: 10.1016/j.tjpad.2026.100648 (PMC13487830; doi:10.1016/j.tjpad.2026.100648)
Supplement: Supplementary file 1 [file mmc1.docx]

APPENDIX

**A-1 Quality Control**

All T1-weighted anatomical scans underwent manual inspection using an MRI viewer (ITK-SNAP) for image quality. Scans were rated based on a visual system adapted from Backhausen et al. Briefly, scans were evaluated according to seven criteria, each rated using a four-point scale where 0 = no/minimal artifact, 1 = minor and localized artifact, 2 = substantial and localized or minor and widespread artifact, and 3 = substantial and widespread artifact. Overall quality ratings were based on the maximum score over the seven items. cores of 0–1 were classified as passing without concern (‘PASS’), a score of 2 required additional post-processing quality checks (‘WARN’), and a score of 3 was grounds for rejection (‘FAIL’). Grading criteria included: brain coverage (the entire brain within the field of view), white-matter/grey-matter contrast (clear delineation of tissue boundaries), image sharpness (clearly defined edges of cortex, subcortex and cerebellum), ringing artifact, aliasing/ghosting artifact, zipper or spike artifact, and susceptibility artifact. All participant scans included in this study had overall ratings of 0 or 1 and were thus retained.

**A-2 Structural Data Processing**

The retained T1-weighted scans were processed using FreeSurfer version 7.2.1. The basic “recon-all” program was first used to generate a map of cortical and subcortical segmentations with the Desikan-Kiliany cortical atlas (denoted “aparc+aseg”), including the left and right lateral-ventricle CP segmentations

**A-3 Choroid Plexus Segmentation**

Given evidence that FreeSurfer estimates of CP segmentations are often error-prone, the segmentations were further refined using a previously published automated Gaussian Mixture Model (GMM) based approach, which has been shown to improve volumetric estimates and is widely used (Tadayon et al., 2020).

A Python script (available on GitHub: https://github.com/EhsanTadayon/choroid-plexus-segmentation) was applied to T1-weighted scans that had undergone intensity normalization using FreeSurfer’s nonparametric nonuniform intensity normalization procedure.

The GMM procedure involved four steps: (1) merging the ventricular and CP segmentations; (2) applying a variational GMM with $K=2$ clusters to distinguish CSF from voxels containing CP and/or ventricular-wall tissue; (3) lightly smoothing the latter cluster using a 1-mm full-width-at-half-maximum Gaussian kernel to reduce ventricular-wall signal through blending with neighboring CSF voxels; and (4) reclustering the result using $K=3$ clusters and designating the highest-intensity cluster as CP

Review of the new segmentations showed substantial improvement over initial FreeSurfer segmentations, but minor segmentation errors remained for many scans. Thus, all of the resulting GMM-based segmentations were subsequently manually reviewed and corrected for cases of under-segmentation and over-segmentation using ITK-SNAP.

This involved a single experienced rater, blinded to the clinical/pathological characteristics of each participant, overlaying the GMM-based segmentations on raw T1-weighted images to assess segmentation accuracy and to correct instances of over and/or under-segmentations such as incorrectly labelled non-CP structures or missed “true” CP areas.

Over-segmentation was defined as extension of the mask into the ventricular wall, CSF, choroid fissure, fornix, and/or septum pellucidum; these areas were trimmed back using the “Paintbrush Tool” (Figure A1).

Under-segmentation occurred when areas of the CP were excluded from the mask; these areas were brought into the mask. Additional checks included the removal of isolated spots in the mask, and boundary refinement, i.e., smoothing of jagged mask edges and cleaning up partial-volume regions. Final checks involved full three-dimensional rendering of the CP segmentations and review from multiple angles to identify any potential areas requiring further cleanup.

**Figure A1**: Workflow of Choroid Plexus Segmentation.

In the leftmost panel, the original T1-weighted image is assessed for the CP of the lateral ventricle. Then (middle panel), GMM CP segmentation is overlaid on the original T1w scan to assess segmentation accuracy to anatomical ground truth. Instances of inaccurate segmentation are then manually cleaned up resulting in the final segmentation (rightmost panel).
